# Recurrence of a t(8;21)-Positive Acute Myeloid Leukemia in the Form of a Granulocytic Sarcoma Involving Cranial Bones: A Diagnostic and Therapeutic Challenge

**DOI:** 10.1155/2013/245395

**Published:** 2013-09-12

**Authors:** Ambra Di Veroli, Alessandro Micarelli, Mariagiovanna Cefalo, Eleonora Ceresoli, Daniela Nasso, Laura Cicconi, Simone Mauramati, Fabrizio Ottaviani, Adriano Venditti, Sergio Amadori

**Affiliations:** ^1^Department of Hematology, “Tor Vergata” University, Viale Oxford 81, 00133 Rome, Italy; ^2^Department of Otorhinolaryngology, “Tor Vergata” University, Viale Oxford 81, 00133 Rome, Italy

## Abstract

Granulocytic sarcoma (GS) is a rare extramedullary solid tumor defined as an accumulation of myeloblasts or immature myeloid cells. It can cooccur with or precede the acute myeloid leukemia (AML) as well as following treated AML. The incidence of GS in AML patients is 3–8% but it significantly rises in M2 FAB subtype AML. This variety of AML harbors t(8;21) in up to 20–25% of cases (especially in children and black ones of African origin) and, at a molecular level, it is characterized by the generation of a fusion gene known as RUNX1-RUNX1T1. Approximately 10% of M2 AML patients will develop GS, as a consequence, the t(8;21) and the relative transcript represent the most common cytogenetic and molecular abnormalities in GS. FLT3-ITD mutation was rarely described in AML patients presenting with GS. FLT3 ITD is generally strongly associated with poor prognosis in AML, and is rarely reported in patients with t(8;21). GS presentation is extremely variable depending on organs involved; in general, cranial bones and sinus are very rarely affected sites. We report a rare case of GS occurring as a recurrence of a previously treated t(8;21), FLT3-ITD positive AML, involving mastoid bones and paravertebral tissues.

## 1. Introduction

Granulocytic sarcoma (GS) is a rare extramedullary solid tumor defined according to the 2008 WHO classification as an accumulation of myeloblasts or immature myeloid cells. It can cooccur with acute myeloid leukemia (AML) or precede its diagnosis as well as following previously treated AML as extramedullary isolated manifestation of relapse [1, 2]. The incidence of GS in AML patients is about 3–8%, but it significantly rises in patients diagnosed with an M2 FAB subtype AML. This variety of AML harbors a translocation t(8;21) in up to 20–25% of cases [3]. At a molecular level, such translocation is characterized by the generation of a fusion gene known as RUNX1-RUNX1T1. Approximately 10% of M2 AML patients will develop GS, as a consequence, the t(8;21) and the relative transcript RUNX1-RUNX1T1 represent the most common cytogenetic and molecular abnormalities seen in GS [4]. Rarely, it was described the presence of *FLT3 *internal tandem duplication (ITD) mutation in AML patients presenting with GS. FLT3 ITD is generally strongly associated with poor prognosis in AML and is rarely reported in patients with t(8;21) [5–7]. GS is more frequently reported in children, with black ones of African origin having a significantly higher incidence particularly in association with t(8;21) [8, 9]. GS clinical presentation is extremely variable depending on organs involved; in general, it is reported that cranial bones and sinus are very rarely affected sites. We report a case of GS occurring as a recurrence of a previously treated t(8;21), FLT3-ITD positive AML, involving mastoid bones and paravertebral tissues. 

## 2. Case Report

**Figure 1:**
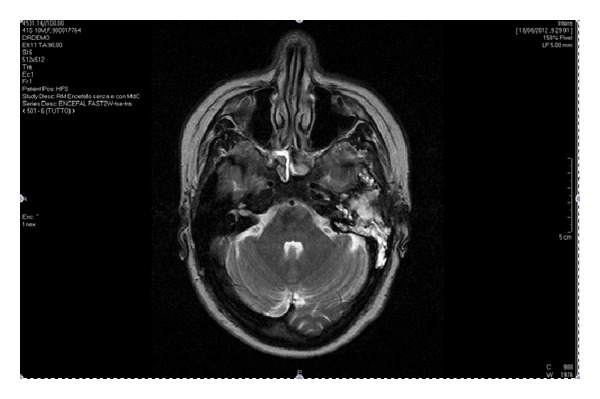
MRI revealing left mastoid cells and the middle ear occupied by a soft tissue density mass.

**Figure 2:**
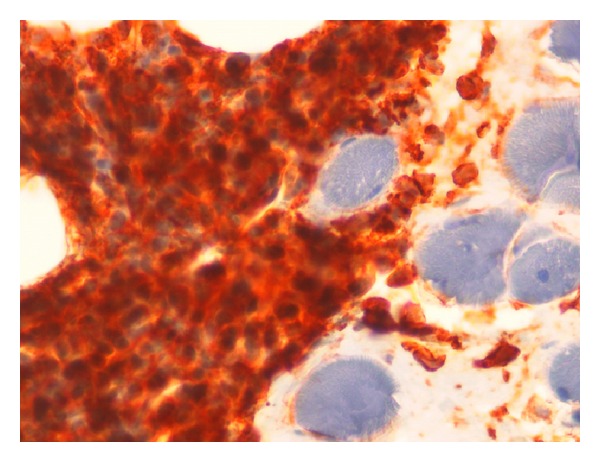
Histological examination of the intraoperative biopsy showing fibroadipose tissue with infiltration of immature myeloid cells (CD 34+).

In July 2011, a 42-year-old female was diagnosed with a M2 FAB subtype AML. Karyotypic and molecular studies revealed that this AML carried t(8;21) and was FLT3-ITD positive. For this reason, the patient received a 3 drug-based induction regimen including daunorubicin (45 mg/m^2^, days 1–3), etoposide (90 mg/m^2^, days 1–5), and cytosine arabinoside (100 mg/m^2^, continuous infusion days 1–7). Upon full hematologic recovery after induction chemotherapy, a morphologic complete remission (CR) was documented by bone marrow aspirate. Consolidation therapy consisted in three courses of chemotherapy associating daunorubicin (45 mg/m^2^, days 1–3) and cytosine arabinoside (450 mg/m^2^, days 1–6). Serial bone marrow (BM) aspirates, performed after each consolidation course, confirmed the condition of morphologic CR; however, quantitative reverse transcriptase polymerase chain reaction (Q-RT-PCR) demonstrated the persistence of RUNX1-RUNX1T1 fusion transcript either after induction or consolidation cycles. In April 2012, after 9 months of continuous CR, the patient was admitted to the emergency department for headache and left ear pains. A cranial magnetic resonance imaging (MRI) indicated the presence of a fluid component in the left mastoid, therefore a diagnosis of otomastoiditis was posed and a broad spectrum antibiotic therapy was instituted. At the same time, a BM reevaluation confirmed the morphological CR and the persistence RUNX1-RUNXT1 transcript. In spite of the administration of antibiotic therapy, symptoms did not resolve, and in May 2012, the patient was again admitted to the emergency department for a VII facial nerve palsy. On clinical examination, the patient had fever and showed left retroauricular swelling and pain, as well as a painful bulging of the upper and posterior walls of the external auditory canal. The eardrum appeared opaque and swollen at the otoscopy. According to the House-Brackmann scale [10] a grade IV left-sided facial palsy was diagnosed. The pure pone audiometry showed a left conductive hearing loss. Computed tomography (CT) with and without i.v. contrast and magnetic resonance imaging (MRI) revealed that the left mastoid cells and the middle ear were occupied by a soft tissue density mass Attic was also filled with soft tissue density mass, but no bone or ossicle destruction was evident (Figure 1). In the assumption that the patient might suffer from atypical left mastoiditis, a surgical approach was planned to clarify diagnosis. After incision of the left-sided retroauricular area, a mastoidectomy was performed. Mastoid, antrum, and aditus were replaced by spongy bone covered with a normal mucosa. Ossicular chain appeared intact, but the caput mallei was englobed in the newly formed bone. After posteroanterior atticotomy, incus and malleus were removed, and posterior tympanotomy was performed. By performing an anteroposterior tympanotomy, exploration of the cage of the middle ear was conducted, and the hyperplastic mucosa was biopsied. Following a canal wall down tympanoplasty (CWDT), a dehiscence upon the second portion of the facial canal was discovered. Therefore, a patch of temporalis muscle fascia was collected and grafted onto facial canal dehiscence, capitulum of the stapes, and promontorium tympany. The graft was then fixed by using scraps of Spongostan, with stitches being arranged in layers. The histological examination of the intraoperative biopsy showed a pluristratified flat epithelium extending over a fibrous tissue. At subepithelial level, there was an infiltration of immature myeloid cells (MPO+, CD 34+, Ki67 = 60–70%), consistent with a diagnosis of AML (Figure 2). Disease staging was completed performing a spine MRI that revealed a solid lumbo-octurator mass. The mass was further explored by a positron emission tomography/computerized tomography (PET/CT) that documented a significant metabolic activity of spine and left mastoid tissue and that incidentally evidenced a focal uterus mass. BM sample analysis confirmed the morphological CR, whereas Q-RT-PCR evaluation still demonstrated persistence of RUNX1-RUNXT1 fusion gene. A diagnostic lumbar puncture documented a cerebrospinal fluid free of AML. Based on the clinical picture, we made a decision to treat the patient with high dose of cytosine arabinoside (3000 mg/m^2^ twice a day, days 1, 3, 5, and 7) associated with an intrathecal injection of cytosine arabinoside (50 mg flat dose) given before systemic therapy and following full hematopoietic resumption to normal blood cell count after chemotherapy. Since a PET/CT scan performed on day 26 after chemotherapy showed a substantial stability of the radiologic picture, we made a decision to carry out a biopsy of the lumbo-octurator mass and a hysterectomy, whereas uterus mass biopsy was excluded due to the very high risk of procedure-related bleeding. The histological examination was consistent with a diagnosis of paravertebral GS and uterine fibroma. Once again bone marrow aspiration confirmed the morphologic CR. Based on the prolonged hematologic CR with a persistent condition of extramedullary disease, the patient was addressed to a program of radiotherapy to irradiate the left mastoid and the spine mass. Radiotherapy was initiated after one month from the end of chemotherapy, and the patient received a total dose of 2200 cGy on the mastoid and of 2800 cGy on the spine mass. In September, before completing the radiotherapy program, a routine blood cell count showed a decrease of platelets count, therefore a BM aspirate was performed. At that stage, the morphologic picture was consistent with a full AML relapse. The patient was then addressed to an additional regimen of chemotherapy in the attempt to induce a second morphologic CR.

## 3. Discussion

Burns described the first case of granulocytic sarcoma (GS)—also called chloroma—in 1823, while the first case of Acute Myeloid Leukemia (AML) associated GS was reported in 1903 by Turk who, therefore, suggested the same origin for both of the tumors [11]. Extramedullary involvement is considered to be an uncommon presentation of AML, although some data suggest that it may be present in up to 30% of patients [12]. GS can develop in different sites as lymphoid organs, bones (orbit, mastoids, etc.), skin, soft tissues, central nervous system, bladder, breast, and uterus [13–16]. Most often, GS happens to anticipate the full AML involvement of bone marrow and/or peripheral blood or can be the initial manifestation of relapse of previously treated AML. On rare occasions, it can occur in the context of a blastic transformation of chronic myeloproliferative disorders or myelodysplastic syndromes [17]. In a recent analysis of 92 adults with de novo GS, 35% and 38% had a concomitant or previous history of AML, respectively [18]. Specific cytogenetic and molecular AML patterns may have a relation with development of GS; in fact, the incidence of GS in previously treated AML is higher among t(8;21)-positive cases. GS associated with translocations t(8;21)(q22;q22)/RUNX1-RUNX1T1 transcript frequently locates in the orbital region in children [9], while those with inv(16) (p13.1q22)/t(16;16)(p13.1;q22) have a high incidence of gastrointestinal tract or breast involvement, especially in the adults [18, 19]. Although to be confirmed, there is also evidence of an association between trisomy 8 positive AML and cutaneous localization of GS [20].

The peculiarity of our case consists in the unusual site of localization of the GS and its association with an AML carrying both t(8;21) and FLT3 ITD mutation. In AML, FLT3-ITD mutations appear to be associated with an increased risk of relapse, short duration of disease free, and overall survival [21, 22]. The incidence of FLT3-ITD mutations is the highest among AML patients with normal karyotype, whereas it is very low in AML with t(8;21). Moreover, FLT3 mutations have been extensively investigated in AML, whereas little is known about their incidence in GS. To the best of our knowledge, only one report demonstrated that FLT3 ITD mutations occur in approximately 15% of GS and that the mutation was concomitantly present in BM and sarcoma specimens in the large majority of cases. In this report, no patients are reported to have FLT3-ITD mutations and contemporary mastoid or paravertebral involvement. Based on this, there is very little evidence to speculate about the association between FLT3 ITD mutations and occurrence of GS even why we were not able to demonstrate the presence of FLT3-ITD mutation in the mastoid sarcoma tissue. Following the general trend in the current literature, GS should always be suspected in young [23, 24] as well as in young adult AML patients with symptoms of acute otomastoiditis associated with facial palsy [25]. In our case, the atypical involved sites and the confounding clinical presentation of mastoid localization have probably deferred the diagnosis by focusing the diagnostic process on an infective problem. In the course of disease staging, we also demonstrated a very rare and unusually asymptomatic localization of GS involving paravertebral tissues. Only few cases of spine GS associated or not with AML have previously been reported [26–29]. Due to the versatility of clinical presentation, diagnosis of GS is missed in about 50% of cases when biopsy is not done and immunohistochemistry is not used. A number of studies have been published on the phenotypic profile of GS [30, 31]. Although not specific, CD43, lysozyme, myeloperoxidase, and CD68 antigens are uniformly identified as the most sensitive markers for they are expressed in the large majority of cases; CD34 can be negative in cases with monocytic differentiation [29]. In this view, Hoyer et al. [32] demonstrated that HLA-DR is more sensitive than CD34, whereas CD45 expression can be erratic. Other commonly used myeloid markers include CD33 [32] and CD117 [33]. In the adult population, differential diagnosis should take into account B/T non-Hodgkin lymphomas and carcinomas. 

In this view, physicians should be aware that imaging diagnostic techniques, such as CT and MRI, might not be conclusive so that a definitive diagnosis could be hard to make outside a surgical scenario. Moreover, a complete as possible disease staging, also including PET and/or TB-CT, should always be attempted to identify additional, asymptomatic, extra-medullary localizations. Patients with GS are treated in the same way as patients with AML independently from bone marrow involvement. Infact extra-medullary disease should be considered a systemic disease and treated with a double therapeutic approach including systemic chemotherapy and additional intrathecal chemotherapy in case of SNC involvement or localized radiation therapy to increase the effectiveness on tumor masses. Chemotherapy is considered the best therapeutic option also for patients with pseudomastoiditis, facial palsy, and GS-related hearing loss. Surgery other than biopsy, should be avoided since surgical decompression of the facial nerve and handling of the middle ear ossicular chain offer a worse outcome than an appropriate chemotherapeutic. In our case, GS did not respond to high dose chemotherapy, therefore we made decision to irradiate the persistent mastoid and paravertebral masses considering that the chemotherapy failure might have been caused not only by chemoresistance but also by a problematic vascular access of drugs to the involved sites. In fact, even though chemotherapy plays an important role in the control of bone marrow disease, it could be inefficient to reach extra-medullary sites which are considered “sanctuaries”. In our case, radiotherapy would have also served as a bridge to allogeneic stem cells transplant to prevent bone marrow relapse. Shimizu et al. [34] analyzed the efficacy of allogeneic hematopoietic stem cell transplantation for the management of AML with GS and reported comparable survival for patients with and without GS once provided an optimal control of GS prior to the transplant procedure. In conclusion, the pathogenesis of GS remains to be fully elucidated, and the prognosis remains unfavorable; however an early intervention, including a complete staging of disease, an accurate molecular study, and a timely delivered chemoradiotherapy followed by allogeneic transplant procedure, is crucial to improve survival.
